# Heart Reflex

**Published:** 1901-01-19

**Authors:** 


### HEART REFLEX.

Dr. Albert Abrams,[1] of San Francisco, describes what he terms the " heart reflex," namely, a contraction of the myocardium, which takes place in response to irritation applied to the precordial region. This effect can be observed only with the Roentgen rays, the fluorescent screen being placed near the anterior chest wall. The reflex is especially pronounced in children. If we irritate the skin in the precordial region by vigorous rubbing with a blunt instrument, " a contraction of the myocardium is observed," the cardiac shadow being diminished in area. This contraction is, as a rule, more manifest in the left than in the right ventricle. The contraction is not sudden and of momentary duration; on the contrary, it lasts as a rule not less than two minutes, and continues after the source of irritation is removed. Dr. Abrams regards this heart reflex as pathognomonic in differentiating a dilatation of the heart from a pericardial effusion. The matter, we think, deserves full investigation. It certainly is of interest as confirming the views held by some in regard to the *modus operandi* of the Nauheim baths, namely, that they do not relieve cardiac dilatation merely by producing vascular relaxation, but that their action upon the skin sets up a reflex stimulation of the cardiac muscle, as the result of which the size of the heart cavities is reduced.

[1] Medical Record.

